# Correlation between apparent diffusion coefficient (ADC) and cellularity is different in several tumors: a meta-analysis

**DOI:** 10.18632/oncotarget.17752

**Published:** 2017-05-10

**Authors:** Alexey Surov, Hans Jonas Meyer, Andreas Wienke

**Affiliations:** ^1^ Department of Diagnostic and Interventional Radiology, University of Leipzig, Leipzig, Germany; ^2^ Institute of Medical Epidemiology, Biostatistics, and Informatics, Martin-Luther-University Halle-Wittenberg, Halle (Saale), Germany

**Keywords:** DWI, MRI, ADC, cellularity, tumor

## Abstract

The purpose of this meta-analysis was to provide clinical evidence regarding relationship between ADC and cellularity in different tumors based on large patient data.

Medline library was screened for associations between ADC and cell count in different tumors up to September 2016. Only publications in English were extracted. The Preferred Reporting Items for Systematic Reviews and Meta-Analyses statement (PRISMA) was used for the research.

Overall, 39 publications with 1530 patients were included into the analysis. The following data were extracted from the literature: authors, year of publication, number of patients, tumor type, and correlation coefficients.

The pooled correlation coefficient for all studies was ρ = -0.56 (95 % CI = [−0.62; −0.50]),. Correlation coefficients ranged from *ρ* =−0.25 (95 % CI = [−0.63; 0.12]) in lymphoma to ρ=−0.66 (95 % CI = [−0.85; −0.47]) in glioma. Other coefficients were as follows: ovarian cancer, ρ = −0.64 (95% CI = [−0.76; −0.52]); lung cancer, ρ = −0.63 (95 % CI = [−0.78; −0.48]); uterine cervical cancer, ρ = −0.57 (95 % CI = [−0.80; −0.34]); prostatic cancer, ρ = −0.56 (95 % CI = [−0.69; −0.42]); renal cell carcinoma, ρ = −0.53 (95 % CI = [−0.93; −0.13]); head and neck squamous cell carcinoma, ρ = −0.53 (95 % CI = [-0.74; −0.32]); breast cancer, ρ = −0.48 (95 % CI = [−0.74; −0.23]); and meningioma, *ρ* = -0.45 (95 % CI = [−0.73; −0.17]).

## INTRODUCTION

Diffusion weighted imaging (DWI) is a magnetic resonance imaging (MRI) technique based on measure of water diffusion in tissues [1]. Beside diagnostic potential, DWI

can distinguish malignant from benign lesions [2, 3]. As reported previously, malignant tumors showed lower apparent diffusion coefficient (ADC) values in comparison to benign lesions [2, 3].

According to the literature, DWI can also provide additional information about tissue microstructure [1, 4–6]. Experimental studies showed a strong association between ADC and cell count *in vitro* [4–6]. It has been shown that increase of cell density restricted water diffusion and decreased ADC [5, 6]. However, published data of clinical investigations were inconsistent. While some authors identified significant correlations between ADC and cellularity in different tumor, other did not [7–11]. Moreover, there was a wide spectrum of reported correlation coefficients ranging from 0.1 to -0.79 [7–12]. Furthermore, the number of investigated patients/tumors in most studies was up to 50 [7–12]. Only few reports analyzed relative large collectives ranging from 102 to 138 patients [13–16]. Therefore, the reported data cannot be considered as evident. Overall, these facts question the possibility to use ADC as a surrogate biomarker for cellularity in clinical practice.

The purpose of this meta-analysis was to provide clinical evidence regarding relationship between ADC and cellularity in different tumors based on large patient data.

## RESULTS

**Figure 1:**
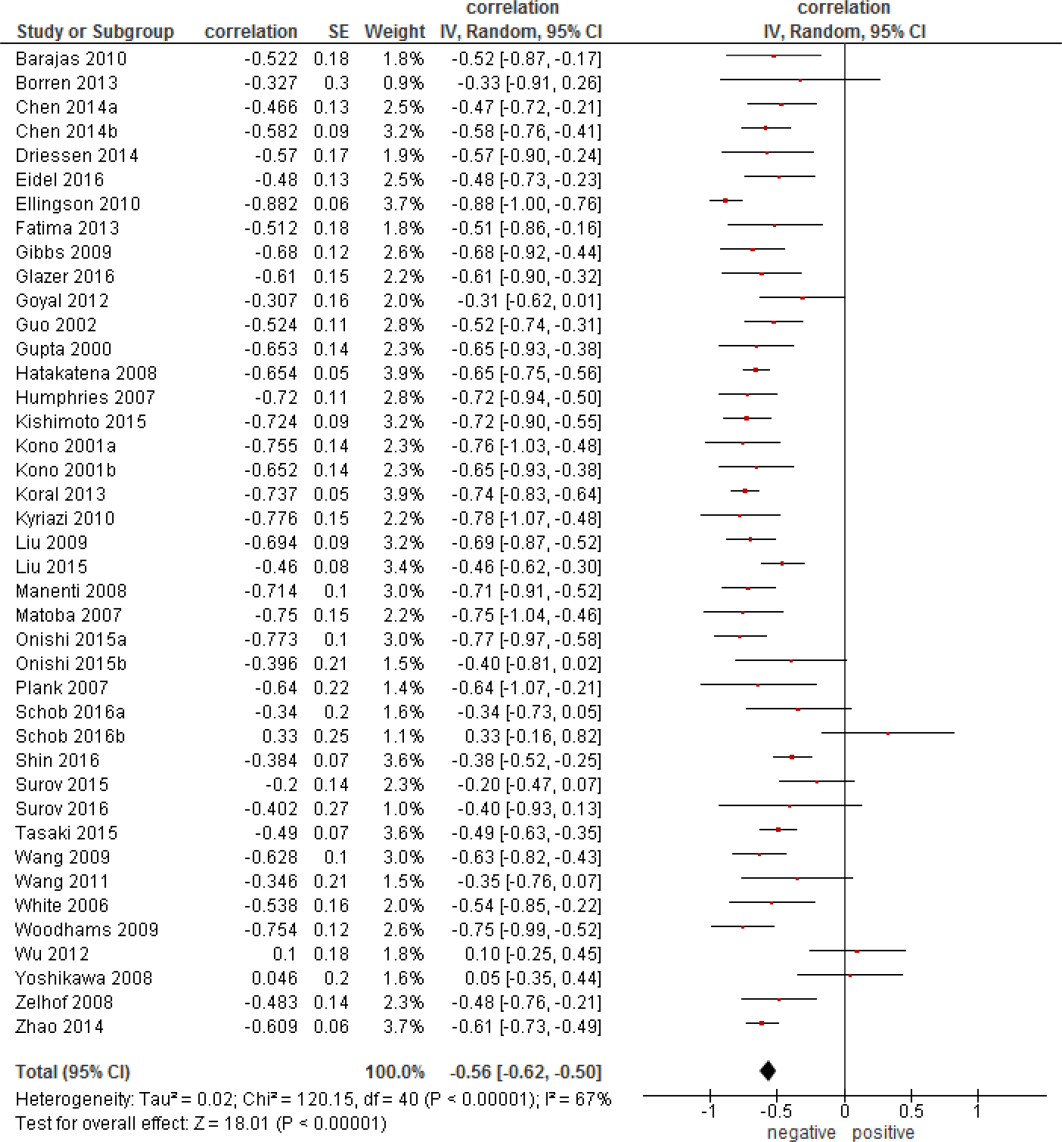
Forest plots of correlation coefficients between ADC_mean_ and cellularity in patients from all involved 39 studies

Overall, the pooled correlation coefficient for all studies (Figure 1) was ρ = −0.56, (95 % CI = [−0.62; −0.50]), heterogeneity τ^2^ = 0.02, (*p* < 0.00001), I^2^ = 67 %, test for overall effect Z = 18.01 (*p* < 0.00001).

**Figure 2:**
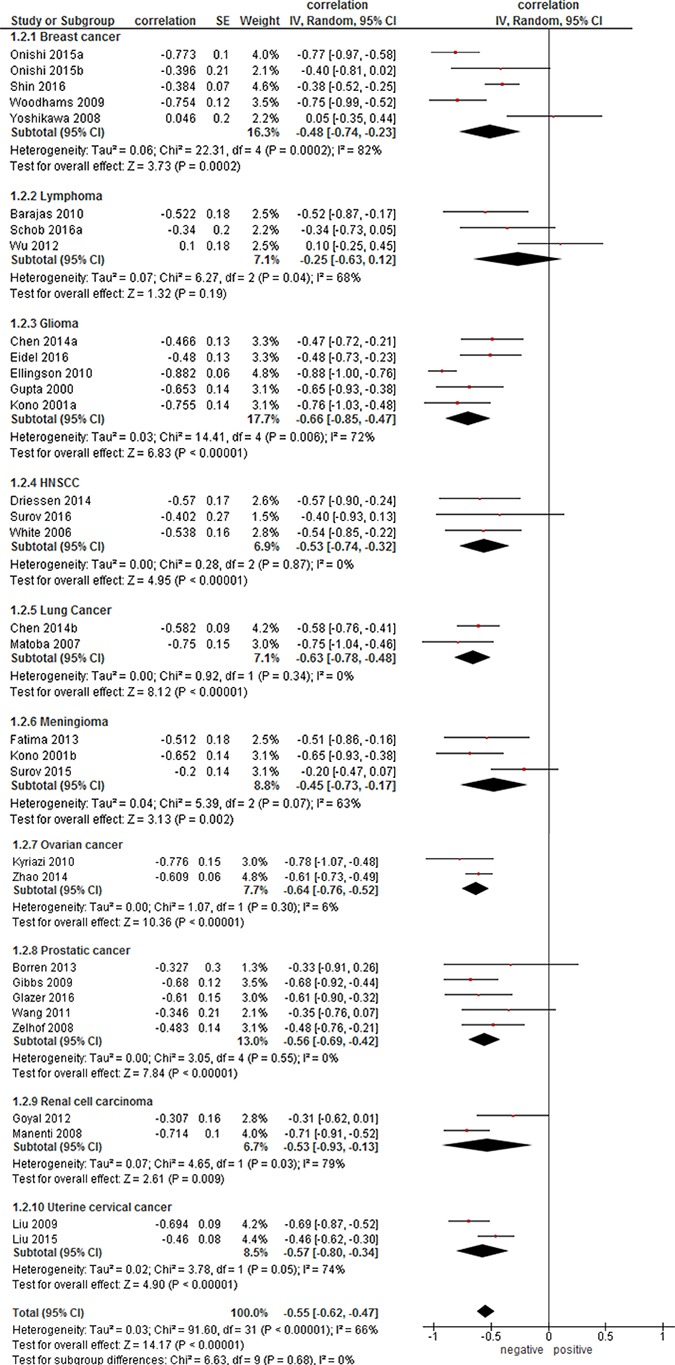
Forest plots of correlation coefficients between ADCmean and cellularity in different primary tumors

On the next step, correlation coefficients for every tumor entities were calculated. For this analysis, only data for primary tumors were acquired (Figure 2). The calculated correlation coefficients ranged from ρ = −0.25 (95 % CI = [−0.63; 0.12]) in lymphoma to ρ = −0.66 (95 % CI = [−0.85; −0.47]) in glioma. Other coefficients were as follows: ovarian cancer, ρ = −0.64 (95% CI = [−0.76; −0.52]); lung cancer, ρ = −0.63 (95 % CI = [−0.78; −0.48]); uterine cervical cancer, ρ = −0.57 (95 % CI = [−0.80; −0.34]); prostatic cancer, ρ = −0.56 (95 % CI = [−0.69; −0.42]); renal cell carcinoma, ρ = −0.53 (95 % CI = [−0.93; −0.13]); head and neck squamous cell carcinoma (HNSCC), ρ = −0.53 (95 % CI = [−0.74; −0.32]); breast cancer, ρ = −0.48 (95 % CI = [−0.74; −0.23]); meningioma, ρ = −0.45 (95 % CI = [−0.73; −0.17]).

## DISCUSSION

The present analysis provides evidence regarding correlation between ADC, in particular ADC_mean_, and cellularity in different tumors based on a large sample.

Previously, numerous studies investigated associations between ADC and cell density in several tumors [7–46]. Overall, most reports showed significant correlations between these parameters [7, 9, 15, 16, 21, 32, 33, 41, 43]. So, Woodhams et al. found a strong inverse correlation ( ρ = −0.75, *p* = 0.001) between ADC and cell count in mucinous breast carcinoma [43]. Based on the reported data, it has been postulated that DWI, namely ADC is an imaging tool to estimate tumor cellularity [43]. However, there were also reports, in which no significant correlations between ADC values and cell count were found [11, 38]. For example, in different lymphomas, the correlation coefficient between cell count and ADC was ρ = 0.1 (*p* = 0.58) [10]. Similar negative results were published for head and neck carcinoma (ρ = −0.418, *p* = 0.201) [39], meningioma ( ρ = −0.20, *p* = 0.164) [38], and breast cancer (ρ = 0.048, *p* = 0.812) [11]. Some previous reports attempted to explain their negative findings by small number of patients [37, 39]. However, another cause of the controversial results in the literature is possible. Presumably, different tumors may have also different associations between ADC and tumor cell count. Our results confirmed this assumption. As seen, ADC showed a moderate inverse correlation with cellularity in the general collective. However, this finding did not apply for each tumor entity, and, therefore, cannot be used in clinical practice. We found that the correlation ADC vs cellularity ranged significantly in different tumors. It was weak in lymphomas, weak-to-moderate in breast cancer and meningiomas, moderate in most investigated epithelial tumors, and strong in gliomas, ovarian cancer, and lung cancer. It is still unclear, why ADC correlates well with cell count in some tumors, whereas in other does not. Presumably, not only cell count, but also other histopathological features, such as extracellular matrix, nucleic areas, ratio stroma/parenchyma, and /or microvessel density may play a role here. In fact, some studies found statistically significant associations between nucleic size and ADC in several lesions [46, 47]. Overall, our findings suggest that ADC does not reflect cellularity in all tumors.

Our analysis also identified another problem. There are no reports regarding associations between ADC and cellularity in most gastrointestinal tumors: esophageal cancer, gastric cancer, colorectal carcinoma, gastrointestinal stromal tumors, hepatocellular carcinoma, pancreatic carcinomas, and gall bladder cancer. Also in malignancies of cutis, such as malignant melanoma, no reports about ADC/cell count could be identified. Except renal cell carcinoma and prostatic cancer, no data exist for urological malignancies. In addition, several tumors involved into the present meta-analysis, for example, HNSCC, renal cell carcinoma, lung cancer, and lymphomas contained small number of patients. This relativizes the calculated results. Finally, for some tumors, such as pancreatic neuroendocrine carcinoma [41], soft tissue sarcomas [15], and thyroid cancer [37], only one report was published, respectively. Therefore, no evident data could be estimated for these entities. Clearly, further researches are needed to investigate possible associations between ADC and cellularity in these tumors. Thereafter, a similar meta-analysis is also needed to prove new data.

In conclusion, different inverse correlations were identified between ADC and cell count in the analyzed tumors. ADC correlated strongly with cell count in gliomas, followed by ovarian cancer, and lung cancer. Therefore, in these tumors, ADC can be used as an imaging marker to estimate cellularity. Moderate inverse correlations were identified between ADC and cell count in prostatic cancer, renal cell carcinoma, uterine cervical cancer, and head/neck squamous cell carcinomas.

Furthermore, weak-to-moderate correlations were found in breast cancer and meningioma. This finding relativizes the possibility of ADC use to predict cellularity in these tumors. Finally, weak correlation was identified in lymphomas. Therefore, ADC cannot be used as a cellularity biomarker in this entity.

No evident data can be provided to date for other malignancies.

## MATERIALS AND METHODS

### Data acquisition and proving

MEDLINE library was screened for associations between ADC and cell count in different tumors up to September 2016. The following search words were used: “DWI or diffusion weighted imaging or diffusion-weighted imaging or ADC or apparent diffusion coefficient AND cellularity or cell density or cell count or cell number”. Only publications in English were extracted. The Preferred Reporting Items for Systematic Reviews and Meta-Analyses statement (PRISMA) was used for the research [48].

After exclusion of duplicates, a total of 494 publications was identified. These reports were involved into the further analysis. For this work, only data regarding ADC_mean_ derived from diffusion weighted imaging (DWI) were acquired. Papers which did not contain correlation coefficients between ADC and cell count were excluded. In addition, data retrieved from diffusion tensor imaging and other DWI parameters, such as D, ADC_max_, and ADC_min_ were also excluded. Finally, we excluded experimental animals and *in vitro* studies. Overall, 455 publications were excluded. Therefore, the present analysis comprises 39 publications with 1530 patients [7–46]. The following data were extracted from the literature: authors, year of publication, number of patients, tumor type, and correlation coefficients. Most frequently, different breast, followed by several brain tumors, uterine sarcomas, uterine cervical cancer, prostatic cancer, and ovarian cancer were reported (Table 1). Other tumors were rarer.

**Table 1:** Patients involved into the study

Diagnosis	*n*	%
**Different breast tumors**	402	26.28
**Different brain tumors**	318	20.78
**Uterine muscle sarcoma**	134	8.76
**Uterine cervical cancer**	130	8.50
**Prostatic cancer**	119	7.78
**Ovarian cancer**	110	7.19
**Lymphoma**	71	4.64
**Lung cancer**	69	4.51
**Renal cell carcinoma**	59	3.86
**HNSCC**	48	3.14
**Endometrial cancer**	30	1.96
**Pancreatic neuroendocrine tumor**	18	1.18
**Thyroid cancer**	14	0.92
**Spinal epidural tumors**	8	0.52
**Total**	**1530**	**100**

HNSCC, head and neck squamous cell carcinoma

### Meta-analysis

The methodological quality of the 39 included studies was independently checked by two observers (A.S. and H.J.M.) using the Quality Assessment of Diagnostic Studies (QUADAS) instrument according to previous descriptions [49, 50]. The results of QUADAS proving are shown in Table 2.

**Table 2:** Methodological quality of the involved 39 studies according to the QUADAS criteria

	Yes (%)	No (%)	Unclear (%)
**Patient spectrum**	24 (61.54)	8 (20.51)	7 (17.95)
**Selection criteria**	25 (64.10)	10(25.64)	4(10.26)
**Reference standard**	39 (100)		
**Disease progression bias**	39 (100)		
**Partial verification bias**	39 (100)		
**Differential verification bias**	39 (100)		
**Incorporation bias**	39 (100)		
**Text details**	39 (100)		
**Reference standard details**	39 (100)		
**Text review details**	18 (46.15)	8 (20.51)	13 (33.33)
**Diagnostic review bias**	18 (46.15)	9 (23.08)	12 (30.77)
**Clinical review bias**	38 (97.44)		1 (2.56)
**Uninterpretable results**	38 (97.44)		1 (2.56)
**Withdrawals explained**	38 (97.44)		1 (2.56)

Spearman's correlation coefficient was used to analyze associations between ADC_mean_ and cell count. The reported Pearson correlation coefficients in some publications were converted into Spearman correlation coefficients as reported previously [51].

The meta-analysis was undertaken by using RevMan 5.3. Heterogeneity was calculated by means of the inconsistency index I^2^[52, 53]. In a subgroup analysis studies were stratified by tumor type. Furthermore, DerSimonian and Laird random-effects models with inverse-variance weights were used without any further correction [54].
